# Supplementary figures and images for: Is the lady's-slipper orchid (Cypripedium calceolus) likely to shortly become extinct in Europe?—Insights based on ecological niche modelling
Source: PLoS One. 2020 Jan 31;15(1):e0228420. doi: 10.1371/journal.pone.0228420 (PMC6993984; doi:10.1371/journal.pone.0228420)

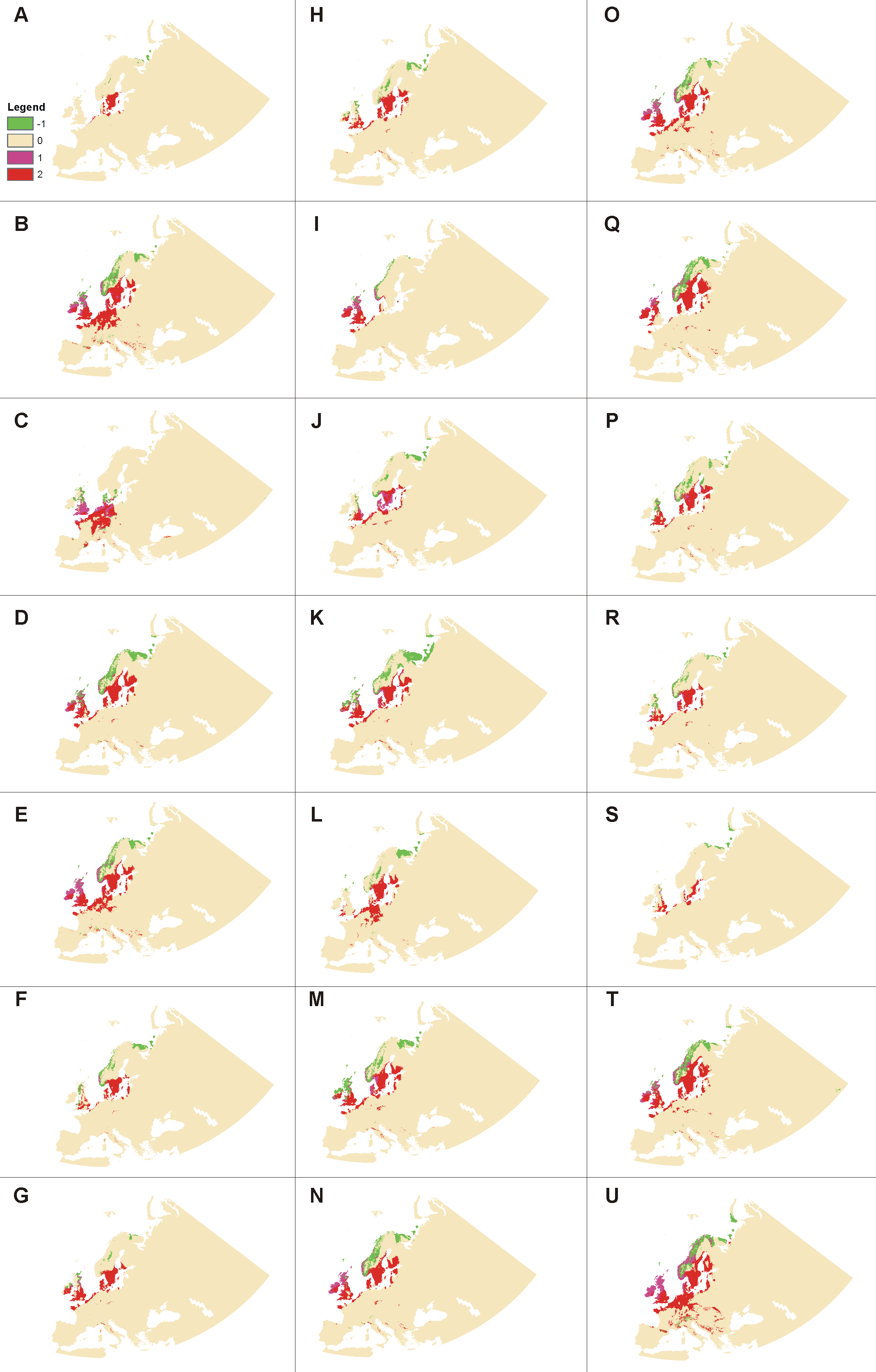

Supplement: S1 Fig — Andrena carantonica (A), Andrena cineraria (B), Andrena flavipes (C), Andrena fucata (D), Andrena haemorrhoa (E), Andrena helvola (F), Andrena nigroaenea (G), Andrena praecox (H), Andrena scotica (I), Andrena tibialis (J), Chrysotoxum festivum (K), Colletes cunicularius (L), Halictus tumulorum (M), Lasioglossum albipes (N), Lasioglossum calceatum (O), Lasioglossum fratellum (Q), Lasioglossum fulvicorne (P), Lasioglossum morio (R), Lasioglossum quadrinotatum (S), Nomada panzeri (T), Syrphus ribesii (U). -1 = range expansion, 0 = no occupancy (absence in both), 1 = no change (presence in both), 2 = range contraction. (TIF) [file pone.0228420.s003.tif]

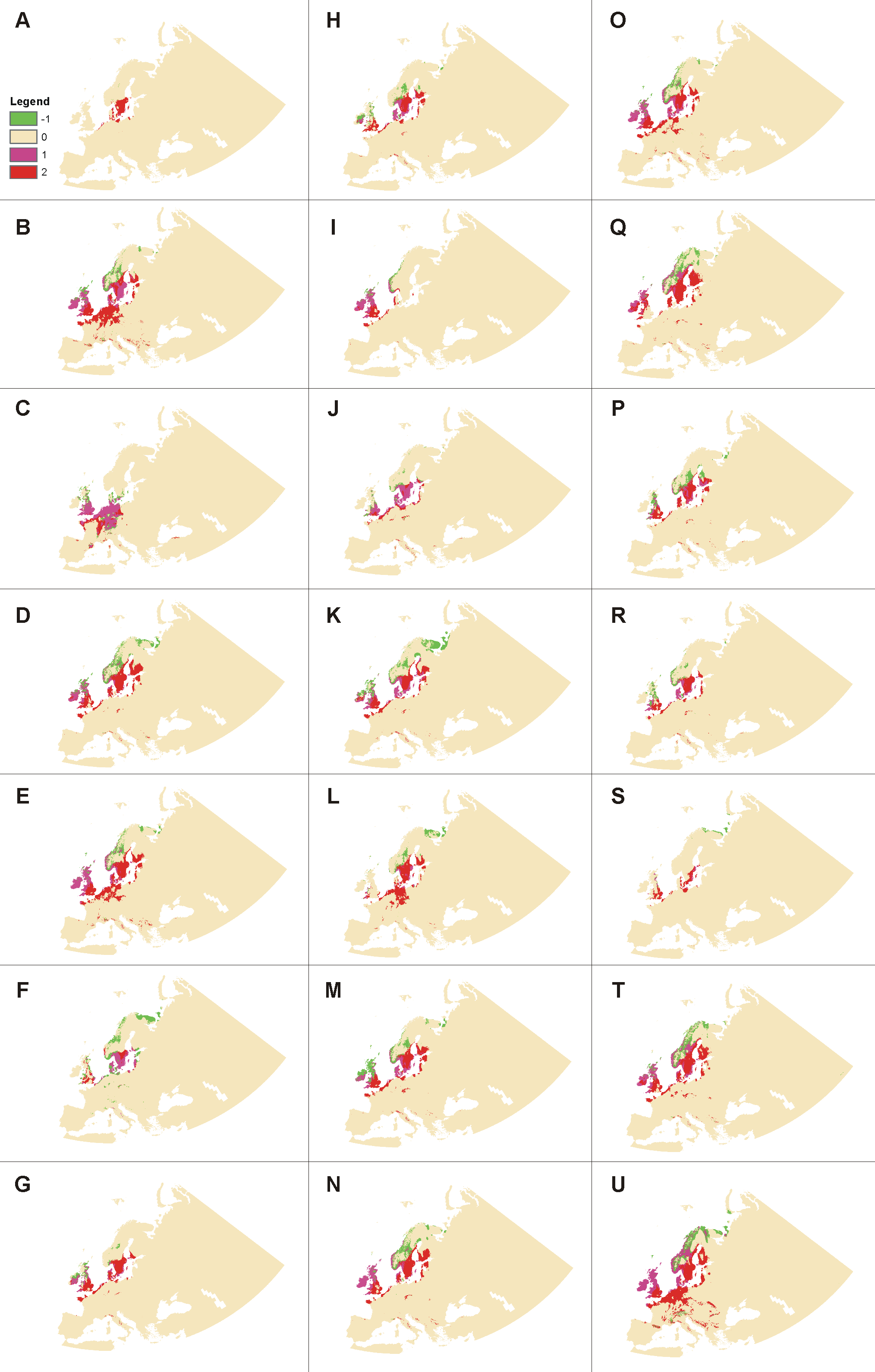

Supplement: S2 Fig — Andrena carantonica (A), Andrena cineraria (B), Andrena flavipes (C), Andrena fucata (D), Andrena haemorrhoa (E), Andrena helvola (F), Andrena nigroaenea (G), Andrena praecox (H), Andrena scotica (I), Andrena tibialis (J), Chrysotoxum festivum (K), Colletes cunicularius (L), Halictus tumulorum (M), Lasioglossum albipes (N), Lasioglossum calceatum (O), Lasioglossum fratellum (Q), Lasioglossum fulvicorne (P), Lasioglossum morio (R), Lasioglossum quadrinotatum (S), Nomada panzeri (T), Syrphus ribesii (U). -1 = range expansion, 0 = no occupancy (absence in both), 1 = no change (presence in both), 2 = range contraction. (TIF) [file pone.0228420.s004.tif]

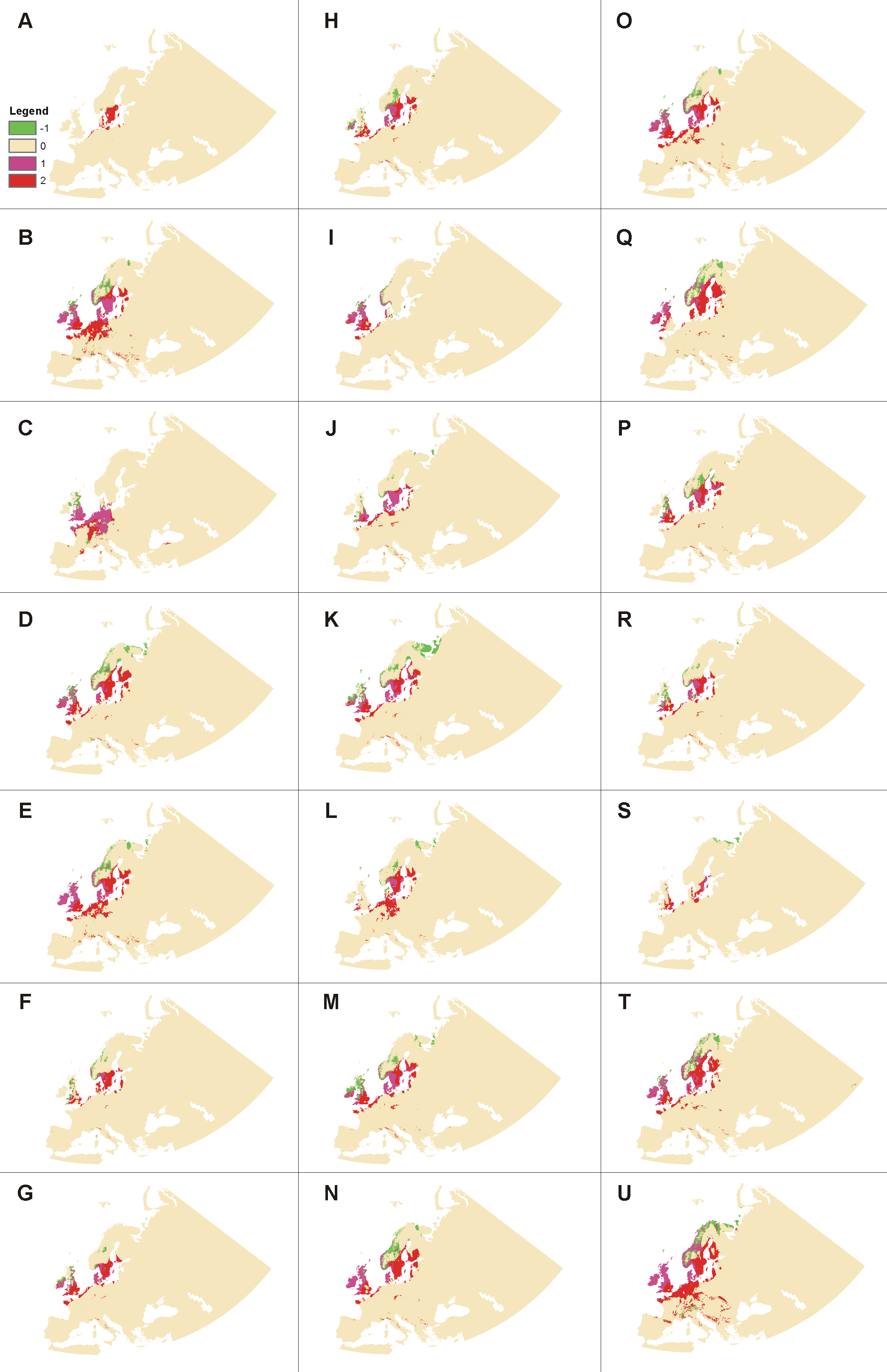

Supplement: S3 Fig — Andrena carantonica (A), Andrena cineraria (B), Andrena flavipes (C), Andrena fucata (D), Andrena haemorrhoa (E), Andrena helvola (F), Andrena nigroaenea (G), Andrena praecox (H), Andrena scotica (I), Andrena tibialis (J), Chrysotoxum festivum (K), Colletes cunicularius (L), Halictus tumulorum (M), Lasioglossum albipes (N), Lasioglossum calceatum (O), Lasioglossum fratellum (Q), Lasioglossum fulvicorne (P), Lasioglossum morio (R), Lasioglossum quadrinotatum (S), Nomada panzeri (T), Syrphus ribesii (U). -1 = range expansion, 0 = no occupancy (absence in both), 1 = no change (presence in both), 2 = range contraction. (TIF) [file pone.0228420.s005.tif]

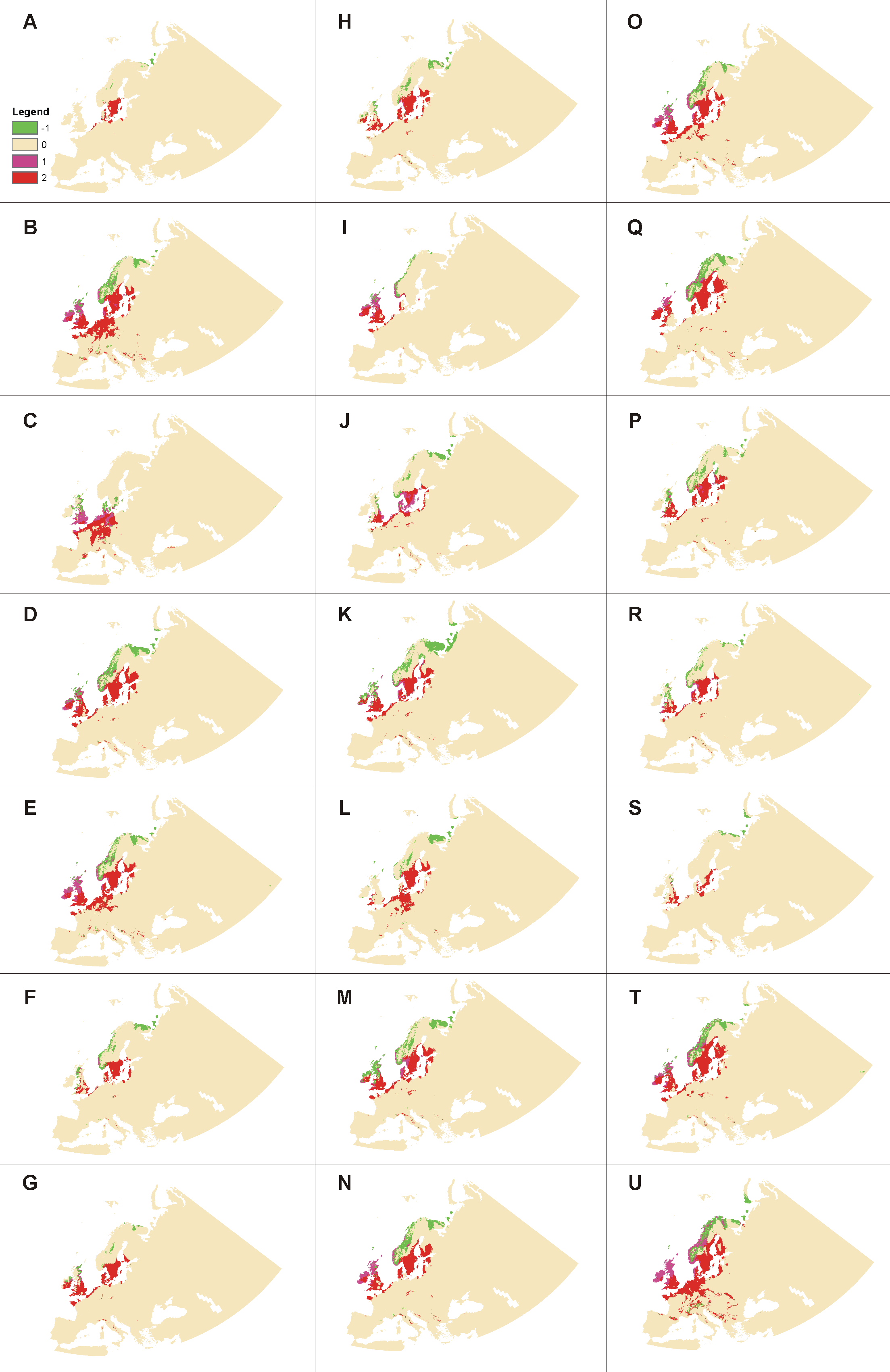

Supplement: S4 Fig — Andrena carantonica (A), Andrena cineraria (B), Andrena flavipes (C), Andrena fucata (D), Andrena haemorrhoa (E), Andrena helvola (F), Andrena nigroaenea (G), Andrena praecox (H), Andrena scotica (I), Andrena tibialis (J), Chrysotoxum festivum (K), Colletes cunicularius (L), Halictus tumulorum (M), Lasioglossum albipes (N), Lasioglossum calceatum (O), Lasioglossum fratellum (Q), Lasioglossum fulvicorne (P), Lasioglossum morio (R), Lasioglossum quadrinotatum (S), Nomada panzeri (T), Syrphus ribesii (U). -1 = range expansion, 0 = no occupancy (absence in both), 1 = no change (presence in both), 2 = range contraction. (TIF) [file pone.0228420.s006.tif]
