# Supplementary material for: Is the lady's-slipper orchid (Cypripedium calceolus) likely to shortly become extinct in Europe?—Insights based on ecological niche modelling
Source: PLoS One. 2020 Jan 31;15(1):e0228420. doi: 10.1371/journal.pone.0228420 (PMC6993984; doi:10.1371/journal.pone.0228420)
Supplement: Attachment — Submitted filename: Response to Reviewers.docx [file pone.0228420.s007.docx]

Additional Editor Comments (if provided):

Dear Author, your manuscript is interesting and worth publishing but needs more work, make sure in your re-submission to adequately address all issues raised by the reviewer, especially trying to enlarge the data collection following the suggestions.

[Note: HTML markup is below. Please do not edit.]

Reviewers' comments:

Reviewer's Responses to Questions

Comments to the Author

1. Is the manuscript technically sound, and do the data support the conclusions?

The manuscript must describe a technically sound piece of scientific research with data that supports the conclusions. Experiments must have been conducted rigorously, with appropriate controls, replication, and sample sizes. The conclusions must be drawn appropriately based on the data presented.

Reviewer #1: Partly

2. Has the statistical analysis been performed appropriately and rigorously?

Reviewer #1: Yes

3. Have the authors made all data underlying the findings in their manuscript fully available?

The PLOS Data policy requires authors to make all data underlying the findings described in their manuscript fully available without restriction, with rare exception (please refer to the Data Availability Statement in the manuscript PDF file). The data should be provided as part of the manuscript or its supporting information, or deposited to a public repository. For example, in addition to summary statistics, the data points behind means, medians and variance measures should be available. If there are restrictions on publicly sharing data—e.g. participant privacy or use of data from a third party—those must be specified.

Reviewer #1: Yes

4. Is the manuscript presented in an intelligible fashion and written in standard English?

PLOS ONE does not copyedit accepted manuscripts, so the language in submitted articles must be clear, correct, and unambiguous. Any typographical or grammatical errors should be corrected at revision, so please note any specific errors here.

Reviewer #1: Yes

5. Review Comments to the Author

Please use the space provided to explain your answers to the questions above. You may also include additional comments for the author, including concerns about dual publication, research ethics, or publication ethics. (Please upload your review as an attachment if it exceeds 20,000 characters)

Reviewer #1: General comments

An interesting Ms that, in my opinion, is it worth to be published after major revision.

I would strongly suggest to address more carefully the distribution of Cypripedium calceolus and all the references and data-bases that could be used. I had the impression the total the number of presence records could be increased after a more careful verification of all the available sources (for a number of Countries). A number of additional potential databases and refences is provided as an example. I think it is worth to improve the data collection and re-run the model.

I think it is very important to start with the better available distribution dataset, if not the level of uncertainly in the model rises too much. In fact, there are already a number of uncertainties areas due to, e.g.: (1) using a single model and not, for example, ensemble modelling techniques; (2) excluding from the model the future changes in land use and the future distribution of beech forest. A number of studies consider the Fagus sylvatica s.l. as a species sensitive to climatic extremes, especially drought and water deficit, which reduces its competitive advantage over less drought-sensitive species, and this will ultimately result in forest vegetation transformation, (3) including in the model the areas that are not and will not be suitable in the future (this could be achieved with masking le land use such as the urban areas that are not of interest).

In the discussion session I would suggest to discuss the results of the present research also in comparison with the modelling of Cypripedium fasciculatum in US done by ThomasN.Kaye (Population extinctions driven by climate change, population size, and time since observation may make rare species databases inaccurate, - PLoSONE 14(10):e0210378.https://doi.org/10.1371/journal.pone.0210378).

Authors: Corrected.

Minor comments

LL 63-64: “Appendix I of the Convention on the Conservation of European Wildlife and 64 Natural Habitats of Bern Convention” – please rephrase: Convention on the Conservation of European Wildlife and Natural Habitats (Bern Convention);

Authors: Corrected.

L 67: “forms” – types;

Authors: Corrected.

L 161: “Figure 1. Localities of C. calceolus georeferenced in this study” -. This map seems not to take into account the distribution of the species in Italy, in particular in the NW, probably due to the fact that Italy is not enough included in GBIF. I would suggest to have a look at: http://dryades.units.it/floritaly/index.php?procedure=taxon_page&tipo=all&id=8099 and http://www.naturachevale.it/wp-content/uploads/2016/06/Cypripedium-calceolus-L_new.pdf

http://fll-italia.it/UploadDocs/6103_G_Perazza___M__Decarli_Perazza_p_129.pdf

http://www.storianaturale.org/anp/PDF%20ANP/24_2003_Isaja%20Dotti_Le%20Orchidee%20spontanee%20della%20Val%20di%20Susa%20Primi%20dati%20sulla%20distribuzione%20di%20tre.pdf

https://www.naturamediterraneo.com/forum/topic.asp?TOPIC_ID=118207

http://www.fondazionemcr.it/UploadDocs/15_art08.pdf

http://www.isprambiente.gov.it/public_files/direttiva-habitat/Manuale-140-2016.pdf (page 128)

I also would strongly suggest to take into consideration the distribution and the reference in the EuroMed PlantBase at:

http://ww2.bgbm.org/euroPlusMed/PTaxonDetailOccurrence.asp?NameId=48350&PTRefFk=8000000

In addition, detailed distribution records are included in the “Action plan for Cypripedium Calceolus in Europe (Nature and Environment No. 100) (1999), Council of Europe” and in TIIU KULL, Journal of Ecology, 1999, 87, 913-924 (BIOLOGICAL FLORA OF THE BRITISH ISLES) and: http://powo.science.kew.org/taxon/urn:lsid:ipni.org:names:320700-2#distribution-map

- LAZARE, J.-J., J. MIRAIXES & L. VILLAR (1987). Cypripedium calceolus (Orchidaceae) en el Pirineo. Anales Jard. Bol. Madrid43(2): 375-382.;

http://citeseerx.ist.psu.edu/viewdoc/download?doi=10.1.1.602.3796&rep=rep1&type=pdf

https://www.conservacionvegetal.org/wp-content/uploads/publicaciones/Catalogo%20de%20especies%20amenazadas%20en%20Aragon.pdf

Authors: We have already used several sources provided by the Reviewer and these were cited in the previous version of ms (e.g. Lazare et al. 1986, Kull 1999, Devilliers-Terschuren 1999 – Action plan, etc.). We could not use all data suggested by the Reviewer due to the lack of possibility of precise georeferencing. Not all links provided by the Reviewer included specific locations but just general information about occurrence of C. calceolus (e.g. <http://powo.science.kew.org/taxon/urn:lsid:ipni.org:names:320700-2#distribution-map>). However, we included additional 43 Italian records based on:

- Perezza G., Decarli Perezza M. 2002. Cartogrfia orchidee trdentine (COT): Cypripedium calceolus L. e Liparis loeselii (L.) Rich., specie citate nella directiva habitat della CEE.
- <https://www.naturamediterraneo.com>
- Perazza G. 1995. Cartografia della orchidee (Orchidaceae) spontanee in Trentino-Alto Adige (Italia). Ricerca Sull'erbario dell'Universita di firence (FI). Ann. Mus. Civ. Rovereto 11: 231-256.
- Pedrini P., Brambilla M., Bertolli A., Prosse F. 2014. Definizione di "linee guida provinciali" per l’attuazione dei monitoraggi nei siti trentinidella rete Natura 2000. LIFE+T.E.N - Azione A5
- Isaja A., Dotti L. 2003. Le orchidee spontanee della Val di Susa (Piemonte-Italia) primi dati sulla distribuzione di tre orchidee rare: Cypripedium Calceolus L. (1735), Corallorhiza Trifida Chatelain (1760) e Aceras anthropophorum R.Br ex Aiton fil. (1814). Riv. Piem. St. Nat. 24: 205-215.

All models for C. calceolus were run again and all statistics were calculated using new outcomes.

L 368: “suitable habitat” – The modelling do not consider habitats but climate, so I would not use the word “habitat” here; in fact in L 333 the term “niches suitable” is used.

Authors: Corrected.

6. PLOS authors have the option to publish the peer review history of their article (what does this mean?). If published, this will include your full peer review and any attached files.

If you choose “no”, your identity will remain anonymous but your review may still be made public.

Do you want your identity to be public for this peer review? For information about this choice, including consent withdrawal, please see our Privacy Policy.

Reviewer #1: Yes: Giuseppe Brundu

[NOTE: If reviewer comments were submitted as an attachment file, they will be attached to this email and accessible via the submission site. Please log into your account, locate the manuscript record, and check for the action link "View Attachments". If this link does not appear, there are no attachment files to be viewed.]

While revising your submission, please upload your figure files to the Preflight Analysis and Conversion Engine (PACE) digital diagnostic tool, https://pacev2.apexcovantage.com/. PACE helps ensure that figures meet PLOS requirements. To use PACE, you must first register as a user. Registration is free. Then, login and navigate to the UPLOAD tab, where you will find detailed instructions on how to use the tool. If you encounter any issues or have any questions when using PACE, please email us at figures@plos.org. Please note that Supporting Information files do not need this step.
